# Antagonism of Ionotropic Glutamate Receptors Attenuates Chemical Ischemia-Induced Injury in Rat Primary Cultured Myenteric Ganglia

**DOI:** 10.1371/journal.pone.0113613

**Published:** 2014-11-24

**Authors:** Elisa Carpanese, Paola Moretto, Viviana Filpa, Silvia Marchet, Elisabetta Moro, Francesca Crema, Gianmario Frigo, Cristina Giaroni

**Affiliations:** 1 Department of Clinical and Experimental Medicine, University of Insubria, Varese, Italy; 2 Department of Surgical and Morphological Sciences, University of Insubria, Varese, Italy; 3 Department of Internal Medicine and Therapeutics, University of Pavia, Pavia, Italy; University of Patras, Greece

## Abstract

Alterations of the enteric glutamatergic transmission may underlay changes in the function of myenteric neurons following intestinal ischemia and reperfusion (I/R) contributing to impairment of gastrointestinal motility occurring in these pathological conditions. The aim of the present study was to evaluate whether glutamate receptors of the NMDA and AMPA/kainate type are involved in myenteric neuron cell damage induced by I/R. Primary cultured rat myenteric ganglia were exposed to sodium azide and glucose deprivation (*in vitro* chemical ischemia). After 6 days of culture, immunoreactivity for NMDA, AMPA and kainate receptors subunits, GluN_1_ and GluA_1–4_, GluK_1–3_ respectively, was found in myenteric neurons. In myenteric cultured ganglia, in normal metabolic conditions, -AP5, an NMDA antagonist, decreased myenteric neuron number and viability, determined by calcein AM/ethidium homodimer-1 assay, and increased reactive oxygen species (ROS) levels, measured with hydroxyphenyl fluorescein. CNQX, an AMPA/kainate antagonist exerted an opposite action on the same parameters. The total number and viability of myenteric neurons significantly decreased after I/R. In these conditions, the number of neurons staining for GluN_1_ and GluA_1–4_ subunits remained unchanged, while, the number of GluK_1–3_-immunopositive neurons increased. After I/R, -AP5 and CNQX, concentration-dependently increased myenteric neuron number and significantly increased the number of living neurons. Both -AP5 and CNQX (100–500 µM) decreased I/R-induced increase of ROS levels in myenteric ganglia. On the whole, the present data provide evidence that, under normal metabolic conditions, the enteric glutamatergic system exerts a dualistic effect on cultured myenteric ganglia, either by improving or reducing neuron survival via NMDA or AMPA/kainate receptor activation, respectively. However, blockade of both receptor pathways may exert a protective role on myenteric neurons following and I/R damage. The neuroprotective effect may depend, at least in part, on the ability of both receptors to increase intraneuronal ROS production.

## Introduction

The intestine is one of the most sensitive organs to ischemia/reperfusion (I/R) injury which may occur as a consequence of embolism, arterial or venous thrombosis, shock [1], intestinal transplantation, necrotising enterocolitis in the human premature newborn or chronic inflammatory diseases [2], [3]. Damage to the intestine may initially and transiently involve the mucosal layer inducing epithelial shedding, bacterial translocation from the lumen into the gut wall, impairment of nutrient absorption, and prolonged reduction in intestinal blood flow [4], [5]. Increasing evidence is, however, available to suggest that intrinsic neuronal circuitries may be damaged following I/R: some neurons may die, while others may undergo changes lasting for many weeks after the injury [5]–[7]. Since both intestinal motility and secretion are highly dependent upon the activity of intrinsic enteric circuitries, such damage may be at the basis of intestinal dysfunctions associated with an ischemic injury in the gut [8]. Investigations on the effects of I/R injury on enteric neurons have evidenced changes both in the morphology, distribution and function of some neuronal pathways, including nitrergic, glutamatergic and peptidergic (e.g. VIP and SP) transmission [6], [7], [9]–[11]. However, little is known about the molecular mechanism/s underlying such changes. In the central nervous system (CNS), glutamate plays a key role in the neuronal damage following an I/R injury [12]. After ischemia, enhancement of extracellular glutamate concentration causes a sustained activation of NMDA ionotropic receptors leading to a rise of cytoplasmic Ca^++^. The increase of free intracellular Ca^++^ initiates a cascade of metabolic events, including production of toxic reactive oxygen species (ROS), leading to cell death [12]. Disruption of Ca^++^ regulatory mechanisms and generation of ROS have been correlated with motility changes occurring during re-oxygenation after hypoxic insults in the gut [13]. Accumulation of nitrosylated protein aggregates resulting from the reaction between nitric oxide (NO) and ROS has been suggested to participate to degeneration of nitrergic neurons following an *in vivo* I/R damage in the mouse gut [7].

Glutamate represents an enteric neurotransmitter/neuromodulator, selectively concentrated in terminal axonal varicosities from where it can be released after application of an appropriate stimulus [10], [14]. Glutamate ionotropic receptors of the NMDA and AMPA type are abundantly expressed on enteric neurons [15] and participate to the regulation of both motor and secretory functions of the gut [14], [16]. However, as observed in the CNS, overactivation of the intrinsic glutamatergic pathways has deleterious consequences on the enteric nervous system (ENS) [17]. Exposure of isolated myenteric ganglia to high extracellular concentrations of glutamate, mimicking ischemic conditions, induces neuronal death, mainly via NMDA and AMPA/kainate receptor activation [17]. There are studies suggesting that glutamate receptors of the NMDA type may participate to alterations of enteric neurotransmitter pathways after I/R injury leading to gastrointestinal dismotility [10], [11], [18]. In the present study, to further investigate the mechanisms underlying glutamate-mediated neurotoxicity in myenteric neurons following an I/R insult, we evaluated whether ionotropic glutamate receptors of the NMDA and AMPA/kainate type are involved in myenteric neuron cell damage induced by I/R. In particular, the ability of -AP5 and CNQX, NMDA and AMPA/kainate receptor antagonists, respectively, to protect cultures of myenteric ganglia from an *in vitro* chemically-induced ischemic injury followed by reperfusion was investigated.

## Materials and Methods

### Myenteric ganglia cultures

Primary cultures of myenteric ganglia were prepared from adult male rats (Harlan Italy, Correzzana, Monza, Italy), weighing between 200 and 225 g, housed in groups of four under controlled environmental conditions (temperature 22±2°C; relative humidity 60–70%) with free access to a standard diet and water, and maintained at a regular 12/12-h light/dark cycle. Principles of good laboratory animal care were followed and animal experimentation was in compliance with specific national and international laws and regulations. The protocol was approved by the Committee on the Ethics of Animal Experiments of the University of Insubria. Animals were sacrificed by decapitation and approximately 20 cm of the distal small intestine, 5 cm oral to the ileo-caecal junction, were rapidly excised and rinsed with a physiological ice-cold Tyrode's solution (composition (mM): 137 NaCl; 2.68 KCl; 1.8 CaCl_2_.2H_2_O; 2 MgCl_2_; 0.47 NaH_2_PO_4_; 11.9 NaHCO_3_; 5.6 glucose). Longitudinal muscle with attached myenteric ganglia (LMMP) were stripped from the whole intestinal wall without penetrating the gut mucosa, thereby avoiding contamination by faecal material. LMMP segments were put in a cold Ca^2+^ and Mg^2+^ free-Hanks' balanced salt solution (HBSS, Euroclone, Milano, Italy) and mechanically minced with small scissors. Incubation in HBSS containing collagenase II (1.5 mg/ml) and protease (1.25 mg/ml) followed for 25 min at 37°C. Digested preparations were then incubated with Trypsin (1.25 mg/ml) and EDTA (0.01%) for 20 min after which time 50% fetal bovine serum (FBS) was added. Cell suspension underwent two consecutive centrifugations at 1500 rpm for 7 min at 4°C, followed by washes in HBSS. The pellet obtained was diluted in 1ml of Dulbecco's Modified Eagle's Medium (DMEM, Euroclone, Milano, Italy), supplemented with 10% FBS, 0.5% glutamine, 1% penicillin/streptomycin and 50 µg/ml gentamycin sulfate, 10 ng/ml glial derived neurotrophic factor (GDNF) and constantly mixed. Cells were counted with trypan blue assay and 3×10^4^ cells per well were seeded on poly-L-lysin (100 ng/L) pre-coated glass cover slips (12 mm in diameter) in 24-well dishes and grown in an incubator (37°C, 5% CO_2_). Myenteric ganglia cultures were grown up for six days in primary cultures and the medium was changed every day.

### Chemical ischemia and drug treatment

At the sixth day, *in vitro* cell cultures were exposed to a transient *in vitro* ischemic injury followed by reperfusion (I/R). To this purpose, *in vitro* ischemic condition were mimicked by replacing the culture medium with glucose-free DMEM (Gibco, Invitrogen, Monza, Italy) supplemented with 10 mM sodium azide for 5 min at 37°C, a condition termed "chemical ischemia" which mimics the energy depletion occurring during ischemia [19]. The effect of reperfusion was evaluated by substituting the medium with fresh and complete DMEM for 24 hours. The effect of the following antagonists, D(-)-2-amino-5-phosphonopentanoic acid (-AP5), 6-cyano-7-nitroquinoxaline-2,3-dione (CNQX), selective NMDA and AMPA/kainate receptor antagonists, respectively, was evaluated by applying drugs at least 20 min before inducing *in vitro* ischemia.

### Immunocytochemistry

Immunocytochemistry was performed on myenteric neuron cultures in normal metabolic conditions and after I/R. Cells on coverslips were fixed in phosphate buffered saline (PBS) containing 4% formaldehyde for 10 min at 4°C. After blocking aspecific sites with PBS containing 5% normal horse serum (NHS) (Euroclone, Celbio, Milan, Italy) and 0.1% Triton X-100 for 1 h at room temperature, preparations were incubated with optimally diluted primary antibodies. To perform double labelling, primary antibodies were exposed during consecutive incubation times: initially, the primary antibody raised either against GluN_1_ (NMDA receptor subunit, 1∶100 Upstate, Millipore), GluA_1–4_ (AMPA receptor subunits, 1∶100 Santa Cruz Biotechnology, Inc Heidelberg, Germany) and GluK_1–3_ (kainate receptor subunits, 1∶200 Santa Cruz Biotechnology, Inc Dallas, TX) was added overnight at 4°C, then incubation with optimally diluted Alexa Fluor488 (Molecular Probes, Invitrogen, Carlsbad, CA, USA) labelled donkey anti-rabbit secondary antibody followed for 2 h at RT. An anti-human neuronal protein HUC/D (1∶100) biotinylated antibody, used as a neuronal cell marker, was successively added and incubated overnight at 4°C, then 2 h incubations with streptavidin Cy3 (1∶300 Caltag Lab., Burlingame, CA, USA) secondary antibody were performed at RT. Preparations were mounted onto glass slides, using a mounting medium with DAPI (Vectashield, Vector Lab., Burlingame, CA, USA). Specificity of GluN_1_, GluA_1–4_, GluK_1–3_ and HuC/D antibodies was tested by their omission. Neuron counts were made on HuC/D stained cell cultures, digitized by capturing 40X objective microscope 10–15 fields (0.287 mm^2^). The total value was divided by the total image field area considered and expressed as the number of cell bodies/mm^2^. Changes of neuron cell number induced by different antagonist treatments during *in vitro* I/R, were expressed as percentage variation with respect to values of cell bodies/mm^2^ number obtained for each treatment in normal metabolic conditions. Experiments were repeated four times using different batches of cells for the different treatment conditions.

The number of GluN_1_, GluA_1–4_ or GluK_1–3_ immunoreactive neurons that co-localized with HuC/D immunoreactive cell bodies were counted and expressed as percentage of the total number of HuC/D positive neurons (10–15 fields). Fields were taken as random images by a blind observer. Experiments were repeated four times. Photographs were analysed by fluorescence microscopy on a Olympus IX 51 (Olympus Italia, Segrate, Italy) and pictures were processed using Adobe-Photoshop CS2.0 software.

### Cell viability/cytotoxicity assay

Survival of cells on coverslips was assessed utilizing a Live/Dead viability/cytotoxicity kit (Molecular Probes, Invitrogen) containing ethidium homodimer and calcein AM. Ethidium homodimer (1 µM) enters dead cells with damaged membranes and stains the nuclei producing a red fluorescence, whereas calcein AM (2 µM) permeates live cells and, after being esterified in the cytoplasm, yields a green fluorescence. Reagents were diluted, applied to coverslips, incubated for 30–45 min and then examined with a fluorescence microscope (Olympus IX 51). The assay was performed on cell cultures, in normal metabolic conditions, after *in vitro* ischemia and after reperfusion, in the presence and absence of -AP5 (100–500 µM) and CNQX (100–500 µM). Ten fields on each coverslip were analyzed utilizing filter sets for each fluorescent label. Fields were taken as random images by a blind observer. The percentage of live (green) and dead (red) cells was calculated on the total cell count, provided by the sum of live and dead cells. At each count a minimum of 50–100 cells (either dead or alive) was counted. Experiments were performed at least three times for each different cell culture preparation.

### ROS levels in cell cultures

An *in vitro* assay of ROS levels in myenteric neurons has been developed using the cell permeable nonfluorescent probe, 2-[6-(4′-Hydroxy)phenoxy-3H-xanthen-3-on-9-yl]benzoic acid2-[6-(4′-Hydroxy)phenoxy-3H-xanthen-3-on-9-yl]benzoic acid] hydroxyphenyl fluorescein, (HPF; Alexis Biochemicals, Nottingham, UK) [20], [21], that after being *O*-dearylated upon reaction with highly reactive oxygen species (hROS, such as hydroxyl radical and peroxynitrite anions) is retained into the cell cytoplasm, exhibiting green fluorescence. Primary myenteric neuronal cultures were prepared as described and maintained at 37°C in a CO_2_ incubator. Samples were treated with 10 µM HPF for 20 min then maintained under the same conditions for a further 1–4 h, in the presence and absence of -AP5 (100–500 µM) and CNQX (100–500 µM). The first measurement was taken at 20 min, when HPF loading was complete (termed time zero). At 0, 1, 2, 3 and 4 h after HPF addition, images were taken using a fluorescence microscope (Olympus IX 51). Fields were taken as random images by a blind observer. This time scale was used since in preliminary experiments we have shown that in control preparations HPF fluorescence peaked around 180 min after the loading period. A minimum of six images of non-overlapping ganglia containing a total of at least 100 neurons from each sample were analyzed. Neuronal profiles were selected and the neuronal areas within a ganglion were used to measure mean grey value per cell (intensity) (GV/cell). Experiments were performed at least three times for each different cell culture preparation and for each drug treatment. Pictures were processed using Adobe-Photoshop CS2.0 software.

### Statistical analysis

For statistical analysis the GraphPad Instat statistical package (version 5.03; GraphPad software, San Diego, CA, USA) was used. Non-linear regression analysis of the concentration–response curves was performed in order to calculate either EC_50_ or IC_50_ values with 95% confidence limits (CL). Significance was tested by analysis of variance (ANOVA) followed by Tukey's test or by two-way ANOVA, as appropriate. A probability of P<0.05 was taken as significant for all statistical analyses.

### Drugs and materials

D(-)-2-amino-5-phosphonopentanoic acid (-AP5), and 6-cyano-7-nitroquinoxaline-2,3-dione (CNQX), were purchased from Tocris (Bristol, UK). All other reagents were purchased either from Sigma-Aldrich (Milano, Italy).

## Results

### GluN_1_ GluA_1-4_ and GluK_1-3_ receptor expression in cultured myenteric ganglia in normal metabolic conditions and during chemical ischemia and reperfusion

**Figure 1:**
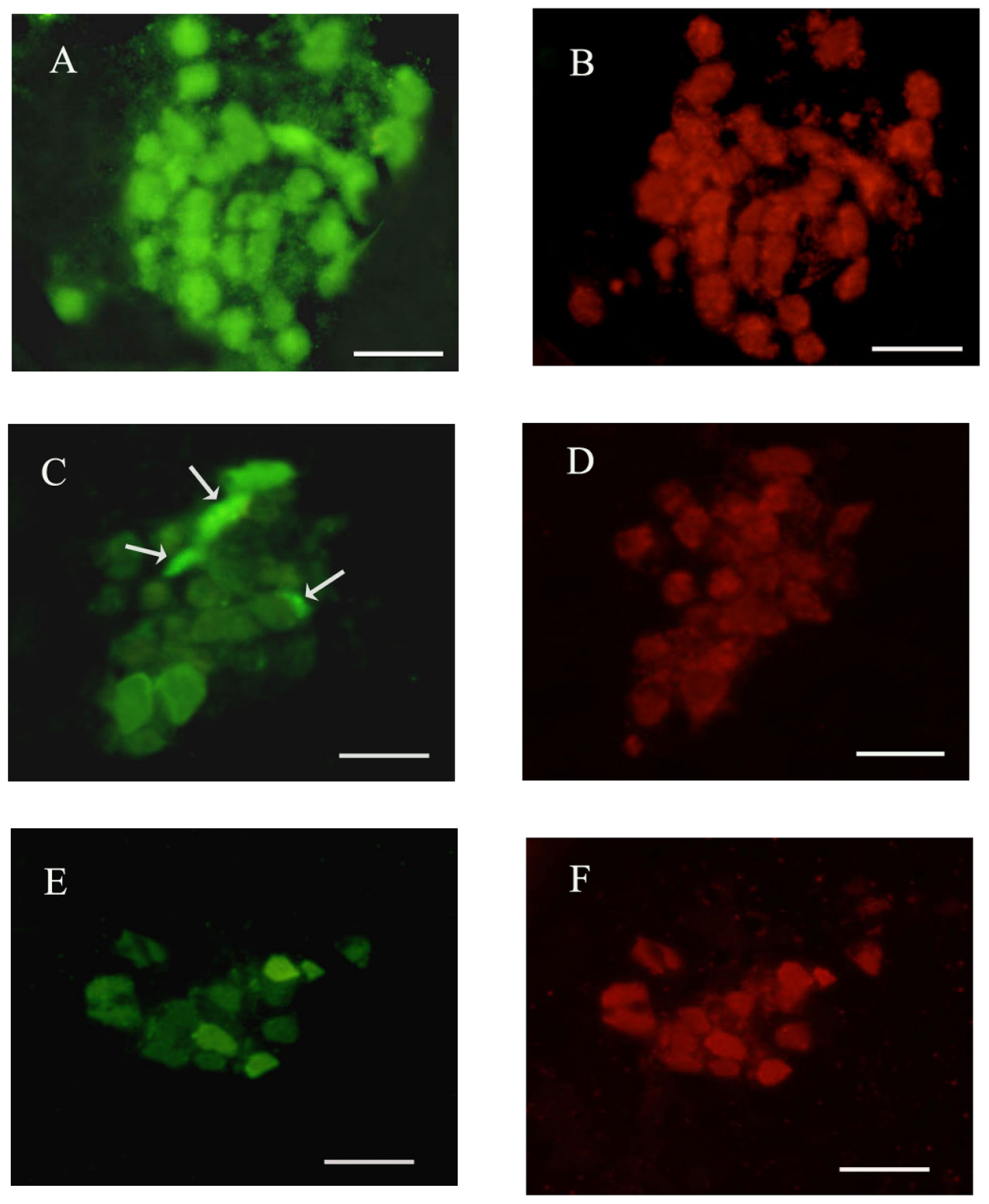
Immunohistochemical co-localization of GluN_1_, GluA_1–4_, GluK_1–3_ with the neuronal marker HuC/D in rat small intestine myenteric ganglia cultures in normal metabolic conditions. GluN_1_ antibody stained the somata of the majority myenteric neurons (A, B). GluA_1–4_ antibody stained the somata of myenteric neurons and the cytoplasm of enteric glial cells (asterisks) (C, D). GluK_1–3_ stained the cytoplasm of myenteric neurons and of enteric glial cells (asterisks). The neuronal marker HuC/D stained the somata of all myenteric neurons (B–D). Bar 10 µm.

Myenteric ganglia cultures contained mainly neurons, glial cells and fibroblasts. Enteric neurons were recognized for the phase-bright appearance and for the positive immunostaining for the neuronal marker HuC/D. On the day of plating neurons appeared small and rounded. After 6 days in culture neurons appeared flattened and larger and started to elongate neurite outgrowth. On day 6 of culture, GluN_1_ immunoreactivity was found in the soma of myenteric neurons and along neurites (Fig 1, panels A–B). GluA_1–4_ antibody stained the cytoplasm of myenteric neurons and enteric glial cells (Fig 1, panels C–D). GluK_1–3_ antibody stained the cytoplasm of myenteric neurons and of enteric glial cells (data not shown) (Fig 1, panels E–F).

**Figure 2:**
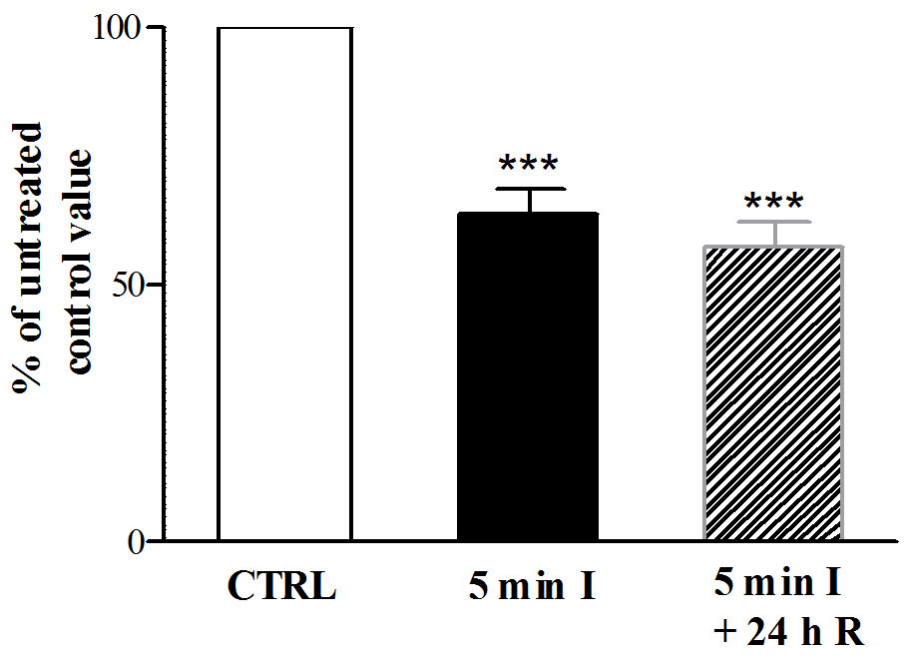
Effect of I/R damage on myenteric neuron number. Bars indicate the percentage variation of the number of rat small intestine myenteric neurons staining for HuC/D after inducing chemical ischemia (black) and after 24 h of reperfusion (slash) with respect to values obtained in normal metabolic conditions (empty). Values are expressed as mean ± SEM of four experiments. Vertical bars indicate SEM ***P<0.001 by one way ANOVA followed by Tukey's test post hoc test.

**Figure 3:**
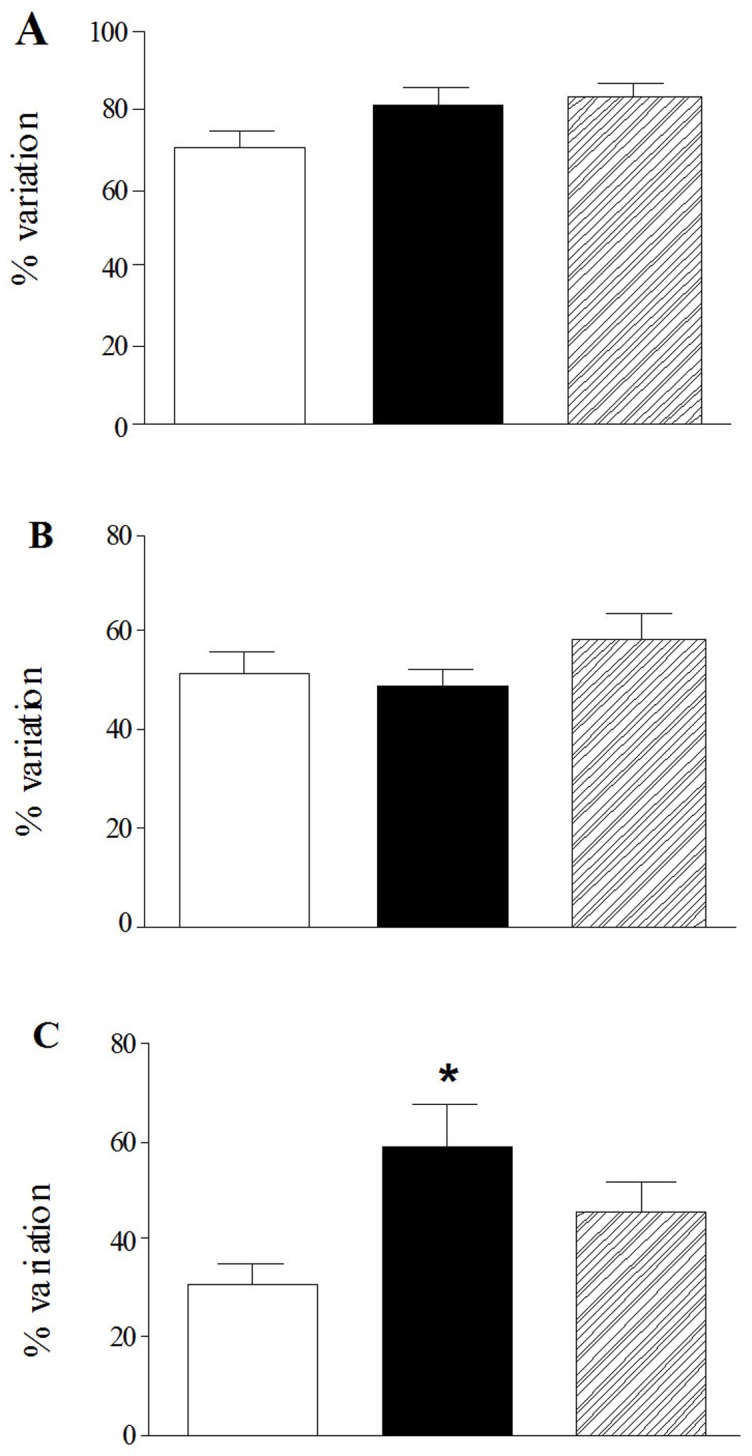
Effect of I/R damage on GluN_1_, GluA_1–4_ and GluK_1_ immunopositive myenteric neuron number. Bars indicate the percentage variation of myenteric neurons staining for GluN_1_ (A), GluA_1–4_ (B) and GluK_1–3_ (C) in rat small intestine myenteric neuron cultures after inducing chemical ischemia (black) and after reperfusion (slash) with respect to values obtained in normal metabolic conditions (empty). Values are expressed as mean ± SEM of three experiments. Vertical bars indicate SEM. *P<0.05 by one way ANOVA followed by Tukey's test post hoc test.

The mean total number of neurons per mm^2^ estimated by counting all HuC/D-immunopositive cells on the coverslips, was 462±72 (n = 4). During both chemical ischemia and reperfusion, this parameter was reduced significantly with respect to control values (36±4.5%, n = 4 during ischemia and 43±4.8%, n = 4 after reperfusion, respectively) (Fig. 2). In control preparations the percentage of myenteric neurons staining for GluN_1_ antibody and for GluA_1–4_ and for GluK_1–3_ antibody was 70.50%±4.63, n = 4; 51.94±3.48%, n = 4 and 30±4.32%, n = 4, respectively. The percentage of myenteric neurons immunopositive for GluK_1–3_ increased after I/R, however values resulted significantly different from control values only after chemically-induced ischemia (59.43±8.32%, n = 4, *P*<0.05 after ischemia; 45.88±5.89%, n = 4 *P*>0.05 after reperfusion). The percentage of myenteric neurons immunopositive for GluN_1_ and GluA_1–4_ was not significantly different after I/R with respect to control values (Fig 3, panels A–B–C).

### Effect of –AP5 and CNQX on the survival of myenteric neurons during chemical ischemia and reperfusion

**Figure 4:**
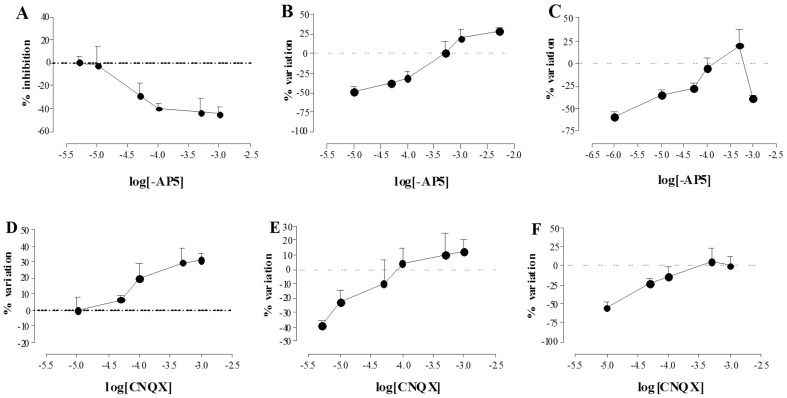
Effect of NMDA and AMPA/kainate receptor blockade on myenteric neuron count after I/R damage. Concentration-response curves represent the effect of -AP5 (A-C) and CNQX (D–F) on the number/mm^2^ of myenteric neurons obtained from rat small intestine after inducing chemical ischemia (B, E) and after reperfusion (C, F) with respect to values obtained in normal metabolic conditions (A, D). Percentage variation of neuron number/mm^2^ is plotted against log molar concentrations of -AP5 (A-C) and CNQX (D–F). Each point represents the mean of 4 experiments. Vertical bars indicate S.E.M.

In normal metabolic conditions, -AP5 concentration-dependently reduced the number of myenteric neurons with an IC_50_ of 21.00 µM with 95% CL of (2.06–216) µM (Fig 4, panel A). After chemical ischemia, –AP5 concentration-dependently increased myenteric neuron number (EC_50_ value: 0.30 µM with a 95% CL of 0.087–1 µM) (Fig 4, panel B). After 24 h of reperfusion, –AP5 in the concentration range of 1–500 µM increased neuron number (EC_50_ of 0.09 µM with a 95% CL of 0.02–0.42 µM). However, in these latter conditions, -AP5, at the concentration of 1 mM, drastically reduced myenteric neuron number (Fig 4, panel C). In normal metabolic conditions, CNQX concentration-dependently increased the number of myenteric neurons with an EC_50_ of 8.20 µM with 95% CL of (8.50–790) µM (Fig 4, panel D). After I/R, CNQX concentration-dependently increased myenteric neuron survival, with EC_50_ values of 19.90 µM with a 95% CL of (1.29–300) µM and 27.00 µM with a 95% CL of (3.10–240) µM, respectively (Fig 4, panel F).

### Effect of –AP5 and CNQX on the viability of myenteric neurons during chemical ischemia and reperfusion

**Figure 5:**
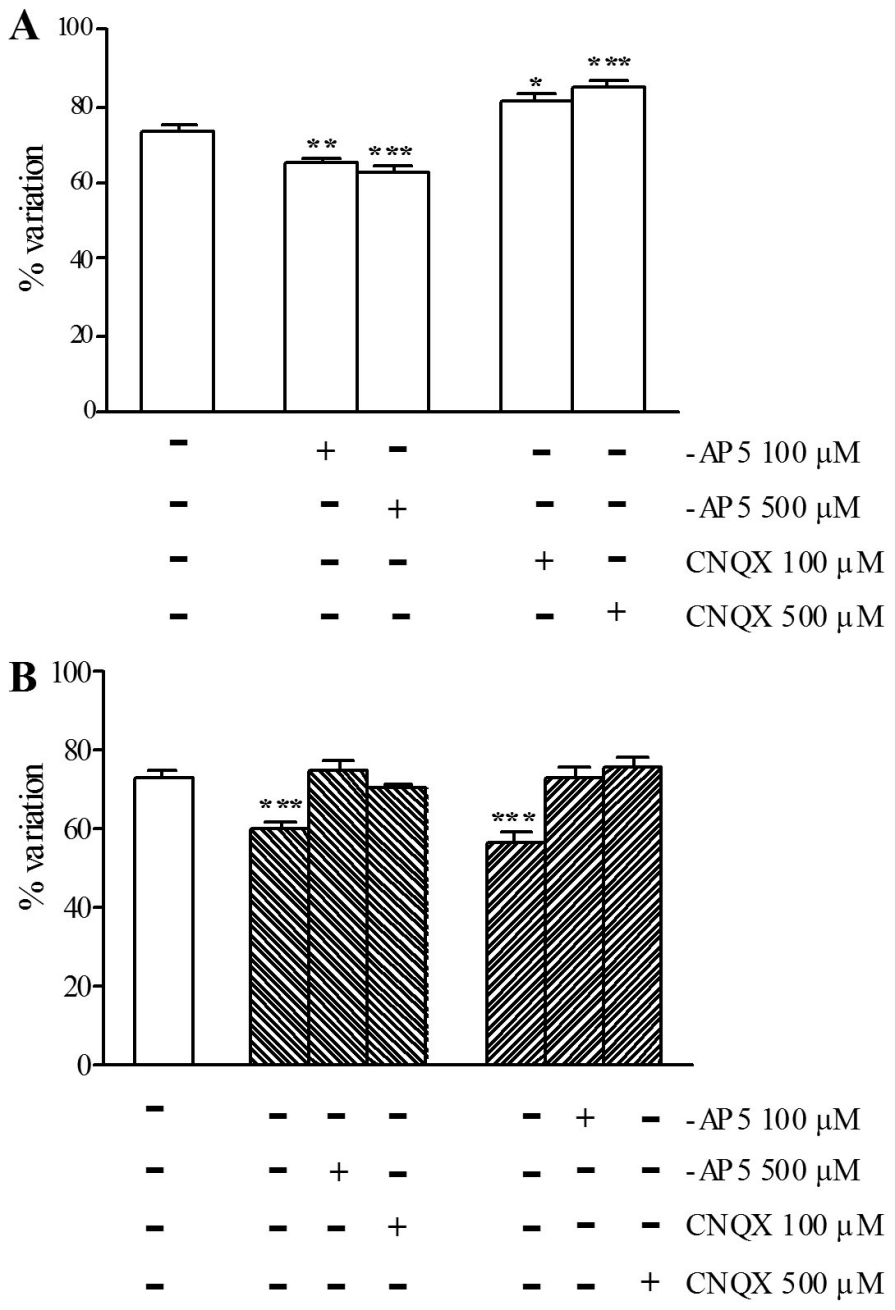
Effect of -AP5 and CNQX on myenteric neuron viability measured by calcein AM permeability after I/R damage. Bars indicate the percentage variation of live cells on the total cell count in the absence and presence of -AP5 and CNQX in normal metabolic conditions (empty bars), after chemical ischemia (backslash) and after reperfusion (slash). Drug treatments are reported at the bottom of each graph. Each point represents the mean of 3 experiments. Vertical bars indicate S.E.M. *P*<0.01 and *P*<0.001 with respect to normal metabolic conditions without drug treatment, by one way ANOVA followed by Tukey's post hoc test.

In normal metabolic conditions, at the concentrations of 100 µM and 500 µM, -AP5 significantly reduced myenteric neuron viability (P<0.01, P<0.001), while CNQX significantly increased (P<0.05, P<0.001) myenteric neuron viability with respect to control preparations (Fig. 5, panel A). Myenteric neuron viability was significantly (P<0.001) reduced with respect to control preparations after *in vitro*-induced I/R (Fig. 5, panel B). In these conditions, at the concentrations of 500 µM and 100 µM, both -AP5 and CNQX increased myenteric neuron viability, reaching values not significantly different from those of control preparations (Fig 5, panel B).

### Effect of –AP5 and CNQX on the ROS production in myenteric neurons during chemical ischemia and reperfusion

**Figure 6:**
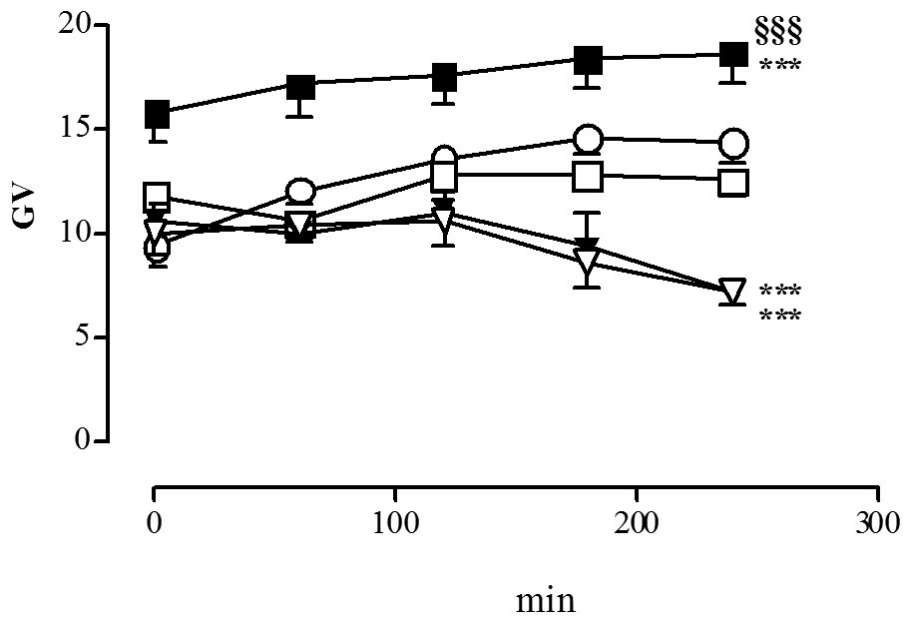
Effect of -AP5 and CNQX on ROS generation in myenteric neurons in normal metabolic conditions. Graphs indicate the extent and timescale of free radical generation (GV =  mean grey value of HPF fluorescence intensity) in enteric neurons, with -AP5 100 µM (□) and 500 µM (▪); CNQX 100 µM (▿) and 500 µM (▾); and in control untreated preparations (○). Each point represents the mean of at least 3 experiments. Vertical bars indicate S.E.M. ***P<0.001 with respect to normal metabolic conditions without drug treatment; ^§§§^ *P*<0.001 with respect to -AP5 100 µM by two-way ANOVA.

HPF fluorescence in control cultures subjected to normal metabolic conditions increased with incubation time, peaking after 3 hours (data not shown). In these conditions, there was no significant difference between the extent of increase in free radical levels in preparations treated with –AP5 100 µM and control preparations, while –AP5 500 µM induced a significant increase of free radical levels compared to control preparations during the whole time-course (Fig. 6, panel A). Incubation with CNQX, 100–500 µM induced a significant decrease of ROS levels with respect to control values which peaked after 4 hours of incubation (Fig. 6, panel B).

**Figure 7:**
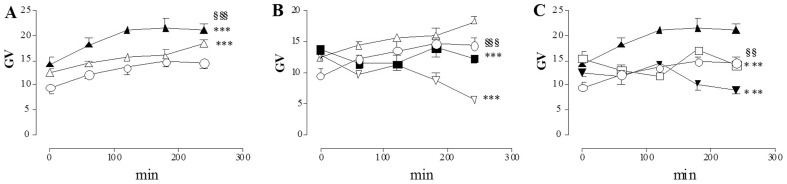
Effect of -AP5 and CNQX on ROS generation in myenteric neurons following I/R damage. Graphs indicate the extent and timescale of free radical generation (GV  =  mean grey value of HPF fluorescence intensity) in enteric neurons, in normal metabolic conditions (○), after chemical ischemia (▵) and after reperfusion (▴) in the absence of any drug (A); in normal metabolic conditions (○), after chemical ischemia (▵) in the presence of -AP5 500 µM (▪) and of CNQX 100 µM (▿) (B); in normal metabolic conditions (○), after reperfusion (▴) in the presence of -AP5 100 µM (□) and of CNQX 500 µM (▾) (C). Each point represents the mean of at least 3 experiments. Vertical bars indicate S.E.M. (A) ***P<0.001 vs normal metabolic conditions, ^$$$^ *P*<0.001 vs *chemical* ischemia conditions; (B) ***P<0.001 vs *chemical* ischemia only, ^§§§^ *P*<0.001 vs CNQX 100 µM; (C) ***P<0.001 vs reperfused preparations only, ^§§^P<0.01 vs CNQX 500 µM by two-way ANOVA.

During both chemical ischemia and reperfusion, neuronal ROS levels significantly increased with respect to control values (Fig. 7, panel A). In both experimental conditions, –AP5 and CNQX at the concentrations of 100 µM and 500 µM significantly reduced free radical generation (Fig. 7, panels B and C). 3–4 after HPF incubation, in I/R conditions, CNQX (100–500 µM) reduced ROS levels to a greater extent than –AP5 (100–500 µM).

## Discussion

Glutamate represents a neurotransmitter/neuromodulator in the ENS, involved in the regulation of both sensory and motor function of the gut [14]. There are, however, evidences suggesting that overactivation of ionotropic receptors of the NMDA and AMPA/kainate type, may exert a neurotoxic effect on myenteric neurons, as observed in the CNS [14]. In the present paper we demonstrate that in primary culture of enteric dissociated ganglia both NMDA and AMPA/kainate receptors are involved in neuronal death following an I/R damage, a condition associated with excessive activation of the glutamatergic transmission [12]. I/R damage was induced by exposing enteric ganglia cultures to a glucose free medium with added sodium azide, to inhibit both oxidative phosphorylation and glycolysis. This treatment has been suggested to closely reproduce all the changes occurring in neurons during ischemia and to be suitable to test potential neuroprotective treatments [19]. After chemically-induced ischemia and during reperfusion with a normal glucose containing medium the number of myenteric neurons, as measured by HuC/D staining, was reduced down to 30–40% with respect to control values. The neurotoxic effect of in vitro induced I/R has been confirmed by the calcein AM/ethidium dead/live assay showing that nearly 40% of myenteric neurons were dead after both treatments. In accordance with our data, sodium azide treatment induced cell death to the same extent in rat cortical neurons [22], [23].

Excessive release of glutamate and the consequent overstimulation of glutamate ionotropic receptors may trigger neuronal death after sodium azide/glucose deprivation-induced I/R [24]–[26]. Our immunohistochemical data indicate that, NMDA, AMPA and kainate subunit receptors are expressed in myenteric neurons after 6 days of culture, both in normal metabolic conditions and after I/R. Immunoreactivity for the GluN_1_ receptor subunit, which represents the ubiquitary and functional subunit of NMDA receptors, was found at a somatodendritic level and along neurites. The antibodies raised against GluA_1–4_ and GluK_1–3_ subunits of AMPA and kainate receptors, respectively, stained the cytoplasm of some neurons and glial cells. Immunoreactivity for the different subunits of NMDA and AMPA receptors has already been detected in whole-mounts LMMPs preparations and in isolated myenteric ganglia of the guinea pig ileum [11], [15], [17]. In a previous study, immunoreactivity for GluK_1–3_ subunits, however, could be found only on submucosal neurons, but not on myenteric neurons [17]. The difference between these observations and our results may rely upon the different antibodies used as well as on different animal models and procedures. The abundance of ionotropic glutamate receptors on myenteric neuron cell culture, in particular of GluN_1_, is in good agreement with immunohistochemistry data obtained in the human colon, guinea pig, rat and mouse ileum [10], [11], [15], [16] and may reflect the functional relevance of enteric glutamatergic pathways in the regulation of the myenteric neuronal networks. In our model, the ability of both -AP5 and CNQX to concentration-dependently increase neuron count and viability following in vitro I/R, strongly suggests that both NMDA and AMPA/kainate glutamate receptors may be involved in the neuronal damage. In agreement with our observations, in rat primary cortical neurons, enhancement of cytosolic Ca^++^ concentration induced by sodium azide treatment was mediated by both NMDA and AMPA/kainate receptor activation [19]. During reperfusion, however, -AP5 increased myenteric neuron number only at the lower concentrations tested, while, at the highest concentration tested (1 mM), the antagonist markedly reduced this parameter, probably reflecting a dualistic action, which may be either neuroprotective or neurotoxic in relation to the concentration used. Interestingly, in normal metabolic conditions, -AP5 concentration-dependently reduced myenteric neuron number and viability. As observed in primary cultures of newborn rat cerebellar granule cells, glutamate may exert a trophic action on isolated myenteric ganglia by mediating Ca^++^ influx via NMDA receptor activation [27]. It is, thus, possible that high concentrations of -AP5 may reduce intracellular Ca^++^ levels necessary for myenteric neuron survival during reperfusion. The NMDA receptor is partially inhibited at physiological concentrations by Mg^++^ (1 mM) and this block is overcome by depolarisation [28]. Sensitivity of myenteric neurons to the NMDA receptor antagonist in the presence of Mg^++^ might be explained considering that, after 6 days of culture, the resting membrane displays a partially depolarized state, similarly to what observed in immature neurons [29]. However, we cannot exclude that in isolated myenteric neurons different GluN subunits may assemble to form a functional heteromeric NMDA receptor displaying low sensitivity to Mg^++^ [30].

Following I/R damage, the number of GluN_1_ and GluA_1–4_, immunopositive neurons did not change with respect to control values, while GluK_1–3_ significantly increased. These data suggest that the neuroprotective action of CNQX may depend, at least in part, upon an increase of GluK_1–3_ receptor availability, while -AP5-mediated neuroprotection may depend upon intracellular mechanism influencing neuronal viability. After *chemical* ischemia, enhancement of extracellular glutamate concentration and sustained activation of NMDA ionotropic receptors induces a rise of cytoplasmic Ca^++^, which on its own initiates a cascade of metabolic events, including production of toxic reactive oxygen species (ROS), leading to cell death [12]. In isolated myenteric neurons both from control and I/R-treated preparations, ROS levels increased with time, reaching a peak at 180 min and remained stable thereafter. Different hypotheses may be put forward to explain the reduced rate of ROS production in the last hour of incubation, including the ability of ROS species to induce antioxidant enzyme transcription via activation of Nrf2, which represents a possible detoxifying feedback mechanism [31]. Under normal metabolic conditions, -AP5 at high concentrations increased ROS levels, supporting the hypothesis that the antagonist may exert a toxic effect on myenteric ganglia in culture. CNQX, on opposite, time-dependently decreased ROS production in control preparations, as indicated by the ability of the drug to reduce HPF fluorescence after 3–4 h of incubation, suggesting that AMPA/kainate receptors may regulate basal ROS production in myenteric neurons, as already observed in the rat brain [32]. This observation is in good agreement with the ability of CNQX to concentration-dependently increase both neuron number and viability in isolated myenteric ganglia. After I/R, ROS levels significantly and progressively increased with respect to control values. During reperfusion, at all time points, HPF fluorescence was higher than after chemically-induced ischemia, suggesting that ROS production in myenteric neurons may raise after restoring normal metabolic conditions. Both NMDA and AMPA/kainate receptors may contribute to such increase, since both -AP5 and CNQX were able to reduce I/R-induced neuronal ROS, although AMPA/kainate receptors seem to retain a more important role, since CNQX displayed a higher efficacy in reducing this parameter [33]–[35]. This data is in line with the increased expression of GluK_1–3_ subunits in myenteric neurons after I/R. In a previous paper, excessive exposure of guinea pig cultured myenteric ganglia to kainic acid has been shown to induce morphological changes and/or disruption in mitochondrial membrane potential, suggesting that excessive activation of kainate receptors may participate to excitotoxicity in the ENS [17]. The mechanism underlying myenteric neuron sensitivity to AMPA/kainate-mediated injury remains to be elucidated. However, we cannot exclude that, as already suggested for some subpopulations of central neurons, this may be largely attributed to the expression of AMPA/kainate channels with high Ca^++^ permeability in myenteric neurons [36].

In our model, ROS production was measured with HPF, a fluorescent dye which is specific for highly reactive oxygen species, such as hydroxyl radicals and peroxinitrites [21], [37]. Since HPF responds primarily to hydroxyl radicals, derived principally from superoxide, our data is indicative of a ROS signal predominantly originating in mithochondrial superoxide. However, the involvement of other oxidative or nitrosative stress species, such as NO, in I/R-induced damage to myenteric neurons in culture cannot be excluded, and requires further investigations [38].

From a functional viewpoint, enhancement of the enteric glutamatergic system may induce alterations of enteric neurotransmitter pathways that might contribute to gastrointestinal dismotility associated with the I/R insult. In the guinea pig ileum, during an ischemic episode, excessive glutamate release and the consequent overstimulation of NMDA receptors has been suggested to be at the basis of changes in the regulation of the cholinergic function and may underlay disturbances in the neuro-effector transmission thus disrupting the endogenous rhythm and coordination of motor activity [10], [11]. In the rat small intestine, glutamate released after in vivo-induced I/R has been suggested to participate to rearrangements in the neurochemical coding of some enteric neurotransmitter pathways leading to the enhancement of nitrergic inhibitory pathways [9]. In this paper we provide direct evidence of a neurotoxic action of both NMDA and AMPA/kainate receptors in myenteric neurons after an I/R injury. Such alterations may depend upon an increase of ROS intracellular levels. Disruption of Ca^++^ regulatory mechanisms and generation of ROS following and I/R damage may have important functional consequences at the intestinal level. In the mouse proximal jejunum, motility changes occurring during re-oxygenation after hypoxic insults in the gut have been correlated with a disruption of the intracellular redox state [13].

## References

[1] Haglund U, Bergqvist D (1999) Intestinal ischemia - the basics. Langenbeck's Arch Surg 384:233–238. 10437610 10.1007/s004230050197

[2] Thornton M, Solomon MJ (2002) Crohn's disease: in defense of a microvascular aetiology. Int J Colorectal Dis 17:287–297. 12172921 10.1007/s00384-002-0408-5

[3] Nowicki PT (2005) Ischemia and necrotizing enterocolitis: where, when, and how. Semin Pediatr Surg 14:152–158. 16084402 10.1053/j.sempedsurg.2005.05.003

[4] Thomson ABR, Keelan M, Thiesen A, Clandinin MT, Ropeleski M, et al 2001;Small bowel review. Diseases of the small intestine. Dig Dis Sci 46:2555–2566. 11768246 10.1023/a:1012782321827

[5] Lindeström LM, Ekblad E (2004) Structural and neuronal changes in rat ileum after ischemia with reperfusion. Dig Dis Sci 49:1212–1222. 15387349 10.1023/b:ddas.0000037815.63547.08

[6] Rivera LR, Thacker M, Castelucci P, Bron R, Furness JB (2009) The reactions of specific neuron types to intestinal ischemia in the guinea pig enteric nervous system. Acta Neuropathol 118:261–270. 19466432 10.1007/s00401-009-0549-5

[7] Rivera LR, Thacker M, Pontell L, Cho HJ, Furness JB (2011) Deleterious effects of intestinal ischemia/reperfusion injury in the mouse enteric nervous system are associated with protein nitrosylation. Cell Tissue Res 344:111–123. 21305320 10.1007/s00441-010-1126-x

[8] Ballabeni V, Barocelli E, Bertoni S, Impicciatore M (2002) Alterations of intestinal motor responsiveness in a model of mild mesenteric ischemia/reperfusion in rats. Life Sci 71:2025–2035. 12175896 10.1016/s0024-3205(02)01966-5

[9] Calcina F, Barocelli E, Bertoni S, Furukawa O, Kaunitz J, et al (2005) Effect of N-methyl-d-aspartate receptor blockade on neuronal plasticity and gastrointestinal transit delay induced by ischemia/reperfusion in rats. Neuroscience 134:39–49. 15939544 10.1016/j.neuroscience.2005.03.052

[10] Giuliani D, Giaroni C, Zanetti E, Canciani L, Borroni P, et al (2006) Involvement of glutamate receptors of the NMDA type in the modulation of acetylcholine and glutamate overflow from the guinea pig ileum during in vitro hypoxia and hypoglycaemia. Neurochem Int 48:191–200. 16290263 10.1016/j.neuint.2005.10.005

[11] Giaroni C, Zanetti E, Giuliani D, Oldrini R, Marchet S, et al (2011) Protein kinase C modulates NMDA receptors in the myenteric plexus of the guinea pig ileum during in vitro ischemia and reperfusion. Neurogastroenterol Motil 23:e91–e103. 21159064 10.1111/j.1365-2982.2010.01644.x

[12] Lau A, Tymianski M (2001) Glutamate receptors, neurotoxicity and neurodegeneration. Pflugers Arch 460:525–542. 10.1007/s00424-010-0809-1 20229265

[13] Bielefeldt K, Conklin JL (1997) Intestinal motility during hypoxia and reoxygenation in vitro. Dig Dis Sci 42:878–884. 9149037 10.1023/a:1018899927786

[14] Kirchgessner AL (2001) Glutamate in the enteric nervous system. Curr Opin Pharmacol 1:591–596. 11757814 10.1016/s1471-4892(01)00101-1

[15] Liu MT, Rothstein JD, Gershon MD, Kirchgessner AL (1997) Glutamatergic enteric neurons. J Neurosci 17:4764–84. 9169536 10.1523/JNEUROSCI.17-12-04764.1997 PMC6573355

[16] Giarossni C, Zanetti E, Chiaravalli AM, Albarello L, Dominioni L, et al (2003) Evidence for a glutamatergic modulation of the cholinergic function in the human enteric nervous system via NMDA receptors. Eur J Pharmacol 476:63–69. 12969750 10.1016/s0014-2999(03)02147-2

[17] Kirchgessner AL, Liu MT, Alcantara F (1997) Excitotoxicity in the enteric nervous system. J Neurosci 17:8804–8816. 9348349 10.1523/JNEUROSCI.17-22-08804.1997 PMC6573082

[18] Cámara-Lemarroy CR, Guzmán-de la Garza FJ, Alarcón-Galván G, Cordero-Pérez P, Fernández-Garza NE (2009) The effects of NMDA receptor antagonists over intestinal ischemia/reperfusion injury in rats. Eur J Pharmacol 621:78–85. 19751722 10.1016/j.ejphar.2009.08.038

[19] Marino S, Marani L, Nazzaro C, Beani L, Siniscalchi A (2007) Mechanisms of sodium azide-induced changes in intracellular calcium concentration in rat primary cortical neurons. Neurotoxicol 28:622–629. 10.1016/j.neuro.2007.01.005 17316809

[20] Setsukinai K, Urano Y, Kakinuma K, Majima HJ, Nagano T (2003) Development of novel fluorescence probes that can reliably detect reactive oxygen species and distinguish specific species. J Biol Chem 278:3170–3175. 12419811 10.1074/jbc.M209264200

[21] Indo HP, Davidson M, Yen HC, Suenaga S, Tomita K, et al (2007) Evidence of ROS generation by mitochondria in cells with impaired electron transport chain and mitochondrial DNA damage. Mitochondrion 7:106–118. 17307400 10.1016/j.mito.2006.11.026

[22] Selvatici R, Previati M, Marino S, Marani L, Falzarano S, et al (2009) Sodium azide induced neuronal damage in vitro: evidence for non-apoptotic cell death. Neurochem Res 34:909–916. 18841470 10.1007/s11064-008-9852-0

[23] He Z, Lu Q, Xu X, Huang L, Chen J, Guo L (2009) DDPH ameliorated oxygen and glucose deprivation-induced injury in rat hippocampal neurons via interrupting Ca^2+^overload and glutamate release. Eur J Pharmacol 603:50–55. 19105952 10.1016/j.ejphar.2008.12.010

[24] Jørgensen NK, Petersen SF, Damgaard I, Schousboe A, Hoffmann EK (1999) Increases in [Ca^2+^]_i_ and changes in intracellular pH during chemical anoxia in mouse neocortical neurons in primary culture. J Neurosci Res 56:358–370. 10340744 10.1002/(SICI)1097-4547(19990515)56:4<358::AID-JNR4>3.0.CO;2-G

[25] Varming T, Drejer J, Frandsen A, Schousboe A (1996) Characterization of a chemical anoxia model in cerebellar granule neurons using sodium azide: protection by nifedipine and MK-801. J Neurosci Res 44:40–46. 8926628 10.1002/(SICI)1097-4547(19960401)44:1<40::AID-JNR5>3.0.CO;2-I

[26] Grammatopoulos TN, Johnson V, Moore SA, Andres R, Weyhenmeyer JA (2004) Angiotensin type 2 receptor neuroprotection against chemical hypoxia is dependent on the delayed rectifier K^+^ channel, Na^+^/Ca^2+^ exchanger and Na^+^/K^+^ ATPase in primary cortical cultures. Neurosci Res 50:299–306. 15488293 10.1016/j.neures.2004.07.010

[27] Burgoyne RD, Graham ME, Cambray-Deakin M (1993) Neurotrophic effects of NMDA activation on developing cerebellar granule cells. J Neurocytol 22:689–695. 7903688 10.1007/BF01181314

[28] Mayer ML, Westbrook GL (1985) The action of N-methyl-D-apartic acid on mouse spinal neurones in culture. J Physiol 361:65–90. 2580984 10.1113/jphysiol.1985.sp015633 PMC1192847

[29] Hanani M, Xia Y, Wood JD (1994) Myenteric ganglia from the adult guinea-pig small intestine in tissue culture. Neurogastroenterol Motil 6:103–118. 22645787 10.1111/j.1365-2982.1994.tb00178.x

[30] Pachernegg S, Strutz-seebohm N, Hollmann M (2012) GluN_3_ subunit-containing NMDA receptors: not just one-trick ponies. Trends Neurosci 35:240–247. 22240240 10.1016/j.tins.2011.11.010

[31] Thompson JW, Narayanan SV, Perez-Pinzon MA (2012) Redox signaling pathways involved in neuronal ischemic preconditioning. Curr Neuropharmacol 10:354–369. 23730259 10.2174/157015912804143577 PMC3520045

[32] Radenovic L, Selakovic V, Kartelija G, Todorovic N, Nedeljkovic M (2004) Differential effects of NMDA and AMPA/kainate receptor antagonists on superoxide production and MnSOD activity in rat brain following intrahippocampal injection. Brain Res Bull 64:85–93. 15275961 10.1016/j.brainresbull.2004.06.001

[33] Iwasaki Y, Ikeda K, Shiojima T, Kinoshita M (1995) CNQX prevents spinal motor neuron death following sciatic nerve transection in newborn rats. J Neurol Sci 134:21–25. 8747838 10.1016/0022-510x(95)00217-6

[34] Carriedo SG, Yin HZ, Sensi SL, Weiss JH (1998) Rapid Ca^2+^ entry through Ca^2+^-Permeable AMPA/Kainate channels triggers marked intracellular Ca^2+^ rises and consequent oxygen radical production. J Neurosci 18:7727–7738. 9742143 10.1523/JNEUROSCI.18-19-07727.1998 PMC6793031

[35] Carriedo SG, Sensi SL, Yin HZ, Weiss JH (2000) AMPA Exposure Induce Mitochondrial Ca^2+^ overload and ROS generation in spinal motor neurons *In Vitro* . J Neurosci 20:240–250. 10627601 10.1523/JNEUROSCI.20-01-00240.2000 PMC6774118

[36] Weiss JH, Yin HZ, Choi DW (1994) Basal forebrain and cholinergic neurons are selectively vulnerable to AMPA/Kainate receptor-mediated neurotoxicity. Neuroscience 60:659–664. 7523984 10.1016/0306-4522(94)90494-4

[37] Thrasivoulou C, Soubeyre V, Ridha H, Giuliani D, Giaroni C, et al (2006) Reactive oxygen species, dietary restriction and neurotrophic factors in age-related loss of myenteric neurons. Aging Cell 5:247–257. 16842497 10.1111/j.1474-9726.2006.00214.x

[38] Giaroni C, Marchet S, Carpanese E, Prandoni V, Oldrini R, et al (2013) Role of neuronal and inducible nitric oxide synthases in the guinea pig ileum myenteric plexus during in vitro ischemia and reperfusion. Neurogastroenterol Motil 25:114–126. 10.1111/nmo.12061 23279126

