# Correction: Antagonism of Ionotropic Glutamate Receptors Attenuates Chemical Ischemia-Induced Injury in Rat Primary Cultured Myenteric Ganglia

**DOI:** 10.1371/journal.pone.0126418

**Published:** 2015-04-13

**Authors:** Elisa Carpanese, Paola Moretto, Viviana Filpa, Silvia Marchet, Elisabetta Moro, Francesca Crema, Gianmario Frigo, Cristina Giaroni

There are errors in the expression of EC50 values in the Results subsection titled “Effect of—AP5 and CNQX on the survival of myenteric neurons during chemical ischemia and reperfusion.” The second sentence should read: After chemical ischemia,—AP5 concentration-dependently increased myenteric neuron number (EC_50_ value: 0.30 mM with a 95% CL of 0.087–1 mM) (Fig 4, panel B). The third sentence should read: After 24 h of reperfusion,—AP5 in the concentration range of 1–500 μM increased neuron number (EC_50_ of 9 μM with a 95% CL of 0.20–420 μM). The fifth sentence should read: In normal metabolic conditions, CNQX concentration-dependently increased the number of myenteric neurons with an EC_50_ of 82 μM with 95% CL of (8.50–790) μM (Fig 4, panel D).
